# Design of Active Vibration Isolation Controller with Disturbance Observer-Based Linear Quadratic Regulator for Optical Reference Cavities

**DOI:** 10.3390/s23010302

**Published:** 2022-12-28

**Authors:** Yuchen Qian, Yong Xie, Jianjun Jia, Liang Zhang

**Affiliations:** 1 Key Laboratory of Space Active Opto-Electronics Technology, Shanghai Institute of Technical Physics, Chinese Academy of Sciences, Shanghai 200083, China; 2 University of Chinese Academy of Sciences, Beijing 100049, China

**Keywords:** microvibration, active vibration isolation, linear quadratic regulator control, disturbance observer

## Abstract

The optical reference cavity in an ultrastable laser is sensitive to vibrations; the microvibrations in a space platform affect the accuracy and stability of such lasers. In this study, an active vibration isolation controller is proposed to reduce the effect of vibrations on variations in the cavity length and improve the frequency stability of ultrastable lasers. Based on the decentralized control strategy, we designed a state-differential feedback controller with a linear quadratic regulator (LQR) and added a disturbance observer (DOB) to estimate the source noise. Experiments were conducted using an active vibration isolation system; the results verified the feasibility and performance of the designed controller. The accelerations along the axis (Z-, X-, Y-) directions were suppressed in the low-frequency band within 200 Hz, and the root-cumulative power spectral densities (PSDs) declined to 1.17 × 10^−5^, 7.16 × 10^−6^, and 8.76 × 10^−6^ g. This comprehensive vibration met the requirements of an ultrastable laser.

## 1. Introduction

As the target accuracy and stability of precision in scientific experiments have improved in recent years, there are more stringent requirements for many experimental instruments in the working environment. Optical clocks [1,2], interferometers [3,4], high-precision microscopes [5,6], long-distance laser communication devices [7,8], and other precision payloads are subject to vibration disturbances, which affect their final accuracy and performance. Space satellites are preferred because they can eliminate complications such as seismic waves and airflow interference, which are prevalent in traditional ground labs. However, space payloads inevitably face microvibration disturbances, generally originating from using flywheels, solar sails, and cryocoolers [9,10,11]. Although low in amplitude, these microvibrations limit further developments toward improving precision in scientific experiments to a large degree [12]. Therefore, research on suppressing these microvibrations using vibration isolation systems has received more attention. Regarding using an optical clock as a precision measurement payload, its performance is inextricably linked to the stability of the optical reference cavity [13]. Since the laser is locked at the resonance frequency of the optical reference cavity, the vibration of the cavity is affected by variations in the cavity length and the final frequency stability, which is pronounced in the low-frequency band. Methods to address the impact of microvibrations on the working of these cavities are vital and need to be studied urgently.

The passive vibration isolation system has received considerable academic attention owing to its reliability. Zhang et al. developed a passive vibration isolation system using multiple coordinated dampers with a Stewart platform. The simulation proved that a vibration isolation effect of 28 dB could be achieved above 100 Hz; the resonance peak amplitude was approximately 4.27 dB [14]. Kamesh et al. designed a passive vibration isolator based on a folded beam that was experimentally validated; it could suppress vibrations above 30 Hz [15,16]. However, owing to their structural features, the passive platform is difficult to further improve vibrations at resonant frequencies. Therefore, semi-active vibration isolation techniques are also widely studied. Memet et al. developed a six-degree-of-freedom parallel isolated platform using a coil-over magnetorheological (MR) damper to reduce the amplification of resonant peaks by varying the damping of the system [17]. Xu et al. used electromagnetic springs to vary the equivalent stiffness of the system and, thus, the resonant frequency [18]. Semi-active vibration isolation reduces power requirements and improves stability. Both passive and semi-active control perform unfavorably for low-frequency, and the suppression effect at the resonant peak needs further improvement. If the performance of these platforms is improved by reducing the resonant frequency, it will lead to insufficient dynamic stiffness and affect the stability of the system [19].

Active vibration isolation technology is usually implemented through sensors and actuators and the corresponding controllers. It provides superior performance in suppressing low-frequency vibrations and flexibility in adjusting the controller to the target [20]. Such features have led to its wide application and development in recent years. Wang et al. designed an active vibration isolation system with multiple degrees of freedom based on three isolators and achieved an average attenuation rate of 94.8% in three directions using sky-hook damping with absolute velocity feedback [21]. Wu et al. designed a separated maglev vibration isolation system, achieving a high-accuracy displacement and attitude control by measuring four groups of two-dimensional position sensors and four laser sources and using proportional derivative (PD) tracking control [22]. Beijen et al. designed an active vibration isolator with a Steward platform; they used the filtered-error least mean squares (FeLMS) algorithm to design the feedforward controller and experimentally demonstrated vibration suppression under the isolator up to 40 dB at 2–300 Hz [23]. Hu et al. used a cascaded proportional–integral–derivative (PID) control design to drive six maglev actuators, achieving vibration suppression up to −22.5 dB in the range of 1–25 Hz [24]. Jiang et al. designed an active vibration isolation system driven by voice coil motors (VCMs) based on a current sheet model and composite nonlinear feedback controller to achieve a suppression effect of approximately −28 dB at 10 Hz [25]. The magnetically suspended inertially stabilized platform developed by Guo et al. achieved the suppression of system rotational torque by a combination of cross-feedback compensation and disturbance observer [26]. A parameter-insensitive reduced-order interference observer was proposed by Hilkert et al. and validated in terms of both disturbance suppression performance and system robustness [27]. Most research on active vibration isolation systems at this stage has been oriented to vibrations in the middle- and high-frequency bands; further research on active vibration isolation systems for low-frequency and low-amplitude microvibrations is still needed.

In this study, a state-differential feedback control strategy with a disturbance observer (DOB)-based linear quadratic regulator (LQR) is proposed for further suppressing microvibrations to meet the vibration isolation requirements of an optical reference cavity. The manuscript is organized into four sections as follows: (1) the problems to be solved in this study, including the demand of the optical reference cavity and the equation of the individual vibration isolation module; (2) the design principle of the controller and the results of the simulation verification; (3) the experimental verification through the active vibration isolation system to confirm the feasibility and effectiveness of the designed controller; and (4) the summary of the main points of the study in the conclusion.

## 2. Problem and System Description

The relationship between the optical reference cavity and verification index under the vibration isolation performance is discussed in this section. Then, the control system is described, and the general framework of the proposed control strategy with a single vibration isolation module is presented as an example.

### 2.1. Vibration Requirements for Optical Reference Cavities

In ultrastable laser systems, the frequency of the laser is obtained by stabilizing the resonance of an optical reference cavity; therefore, the variations in the cavity length of the optical reference cavity are a major factor in determining its frequency stability [28]. Regarding a conventional optical reference cavity, the vibrations along the three axial directions have the greatest effect on cavity deformation; this is the main target of interest in this study. Taking a single direction as an example, the frequency noise caused by vibration can be expressed as [29]:

(1) $$\sigma_{i}=k_{vi}\sqrt{\int_{0}^{\infty }{\left|H_{\gamma }(f)\right|}^{2}S_{i}(f)df}\;(i=x,y,z),$$

where $k_{vi}$ is the vibration sensitivity of the cavity; f is the frequency; $S_{i}(f)$ is the PSD distribution of the vibrations in that direction; and $H_{\gamma }(f)$ is the transfer function of the Allan variance with the measurement average time γ, which is expressed as:

(2) $${\left|H_{\gamma }(f)\right|}^{2}=2\frac{\sin^{4}(\pi f\gamma )}{{\left(\pi f\gamma \right)}^{2}},$$

**Figure 1:**
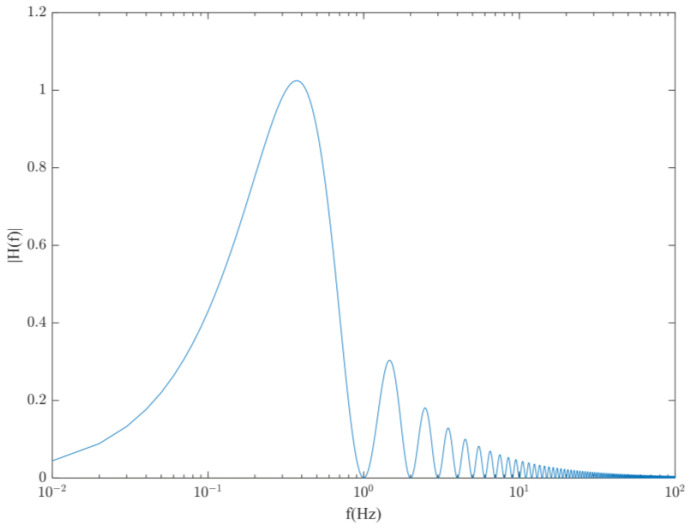
Vibration transfer function for Allan variance for γ=1 s.

The main factors determining the vibration noise are the vibration sensitivity $k_{vi}$ of the cavity and the PSD of the vibrations $S_{i}(f)$, where $k_{vi}$ is determined by the cavity structure, material, and support method. The National Physical Laboratory (NPL) designed a cubic geometry with four supports placed symmetrically about the optical axis in a tetrahedral configuration, and the final measured maximum acceleration sensitivity was 2.5 × 10^−11^ g [30]. However, $k_{vi}$ is typically a frequency-independent constant, and once the cavity is completely designed, further optimization of the process is difficult. The Allan variance transfer function shown in Figure 1 indicates that the effect of the vibration transfer is much higher in the low-frequency band. Therefore, in this study, we focus on reducing the effect of vibration on the frequency stability of the laser by suppressing the PSDs of the three cumulative vibrations along the axial direction.

.

### 2.2. Description of Active Vibration Isolation System

**Figure 2:**
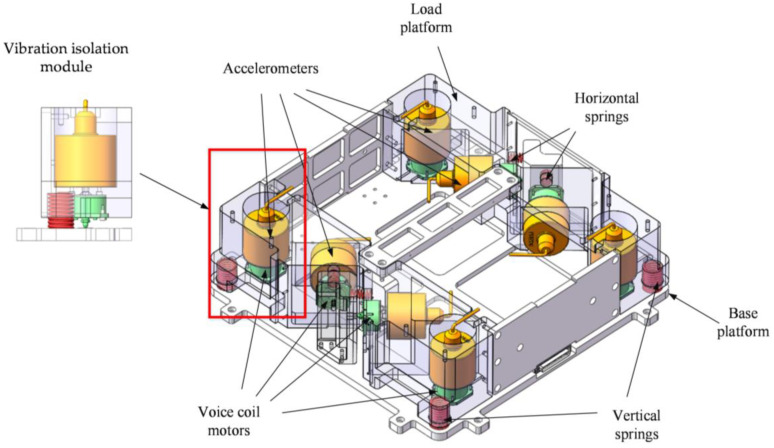
Structure of active vibration isolation system.

The system used in this study was a self-developed active vibration isolation system [31]. The structural arrangement of the active vibration isolation platform (AVIS) is shown in Figure 2. The entire system consisted of a foundation platform, payload platform, and eight vibration isolation modules between them. These modules were arranged orthogonally, with half along the direction of gravity, and the other half along the horizontal direction. Each module contained coaxially mounted accelerometers and VCMs for collecting signals and driving control forces. Structurally, as shown in the enlarged part of Figure 2, they can be considered as a spring-mass-damper system module. The system uses a decentralized control strategy, where each module is controlled independently. In this way, they can be considered as a closed-loop single-input single-output (SISO) system based on sensor measurements and actuator outputs. Each module has the same structure and control loop, differing only in the parameters of the controller.

**Figure 3:**
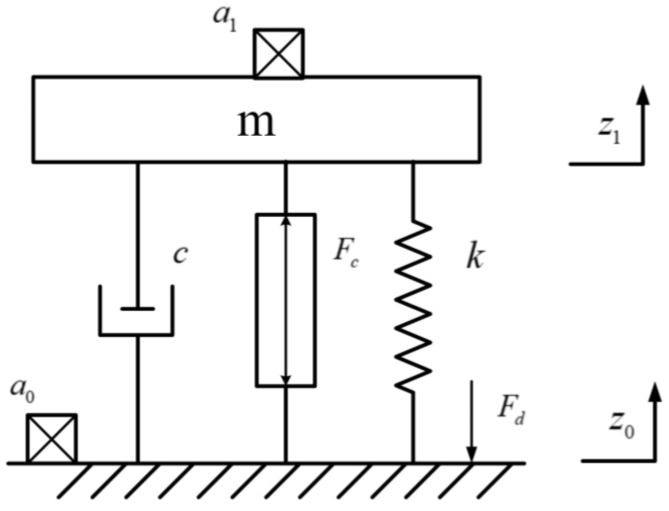
Schematic diagram of the vibration isolation module.

A schematic of a single vibration isolation module is shown in Figure 3. The equivalent stiffness, damping, and mass are k, c, and m, respectively; these form the passive structure of the mass-spring-damper model. The effect of the noise $a_{1}$ transmitted from the base plane noise $a_{0}$ to the payload platform was reduced by applying the active control force $F_{c}$ provided by the VCM; $z_{0}$ and $z_{1}$ were the base platform and payload platform displacements, respectively; and $F_{d}$ could be regarded as a disturbance force from the base platform. The force balance equation is expressed as follows:

(3) $$m{\ddot{z}}_{1}+c{\dot{z}}_{1}+kz_{1}=c{\dot{z}}_{0}+kz_{0}+F_{c}=F_{d}+F_{c},$$

The payload platform was assumed to be subjected to a combination of the disturbance and control forces. In this study, the disturbance force was mainly the vibration of the base platform transmitted by the spring and damping. The control force was mainly the electromagnetic force generated by the VCM, which can be simplified and expressed as:

(4) $$F_{c}=k_{c}I_{c},$$

where $k_{c}$ is the output coefficient of the VCM, and $I_{c}$ is the control current.

The state-space equation of the system can be expressed as:

(5) $$\dot{x}=Ax+B_{1}\omega +B_{2}u,$$

where $x={\left[\begin{array}{cc} z_{1} & {\dot{z}}_{1} \end{array}\right]}^{T}$, and $\omega =[F_{d}]$, $u=[I_{c}]$. The state matrices are represented as follows:

(6) $$A=\left[\begin{array}{cc} 0 & 1\\ -\frac{k}{m} & -\frac{c}{m} \end{array}\right],B_{1}=\left[\begin{array}{c} 0\\ \frac{1}{m} \end{array}\right],B_{2}=\left[\begin{array}{c} 0\\ \frac{kc}{m} \end{array}\right],$$

The active vibration isolation system was controlled by acceleration feedback. Considering the possible drift of the measured signal in the low-frequency band, a state-differential feedback controller with an LQR was designed in this study to calculate the control quantity u. Furthermore, the disturbance force from the base plane was estimated with the assistance of a DOB to ensure the suppression effect and control accuracy of the payload platform acceleration.

## 3. Design of Controller

### 3.1. LQR State-Differential Controller

The state-space representation of an LQR controller is given by:

(7) $$\dot{x}=Ax+B_{2}u,$$

Note that a conventional LQR controller requires full-order state feedback control, which is difficult to realize during the operation of a real system [32]. Particularly, the quantity measured in this study was acceleration. If the state quantity is obtained via direct integration, it tends to cause a drift in the signal. Therefore, LQR state-differential feedback control was used, wherein the controller was obtained by differentiating the state quantities as follows:

(8) $$u=-K\dot{x},$$

Substituting Equation (8) into Equation (7):

(9) $$(I+B_{2}K)\dot{x}=Ax,$$

The cost function is formulated as:

(10) $$J=\int_{0}^{\infty }\left({\dot{x}}^{T}(t)Q\dot{x}(t\right)+u^{T}(t)Ru(t))dt=-x^{T}Px,$$

where Q and R are the weight matrices of the state and control, respectively; and P is a positive definite matrix. Differentiating on both sides’ yields:

(11) $${\dot{x}}^{T}(Q+K^{T}RK){\dot{x}}^{T}={\dot{x}}^{T}Px+x^{T}P\dot{x},$$

Substituting into Equation (11), the algebraic Riccati equation can be obtained as follows:

(12) $$PA^{-1}+PK^{T}B_{2}{}^{T}A^{-1}+A^{-T}P+A^{-T}B_{2}KP+Q+K^{T}RK=0,$$

The above equation can be rewritten as a linear matrix inequality (LMI) using the Schur complement [33]. The specific equation is as follows:

(13) $$\left[\begin{array}{ccc} PA^{-1}+A^{-T}P+A^{-1}B_{2}KP+PK^{T}B_{2}{}^{T}A^{-T} & I & K^{T}\\ I & -Q^{-1} & 0\\ K & 0 & -R^{-1} \end{array}\right]<0,$$

where P is a unique symmetric positive definite solution that satisfies matrix inequality. Defining $Y=P^{-1}$ and W=KY:

(14) $$\left[\begin{array}{ccc} YA^{-1}+A^{-T}Y+A^{-1}B_{2}W+W^{T}B_{2}{}^{T}A^{-T} & Y & W^{T}\\ Y & -Q^{-1} & 0\\ W & 0 & -R^{-1} \end{array}\right]<0,$$

By solving the matrices Y and W, the final controller $K=WY^{-1}$ can be calculated. Moreover, the Lyapunov function was also satisfied by the calculated P value, ensuring the stability of the controller.

### 3.2. Disturbance Observer

**Figure 4:**
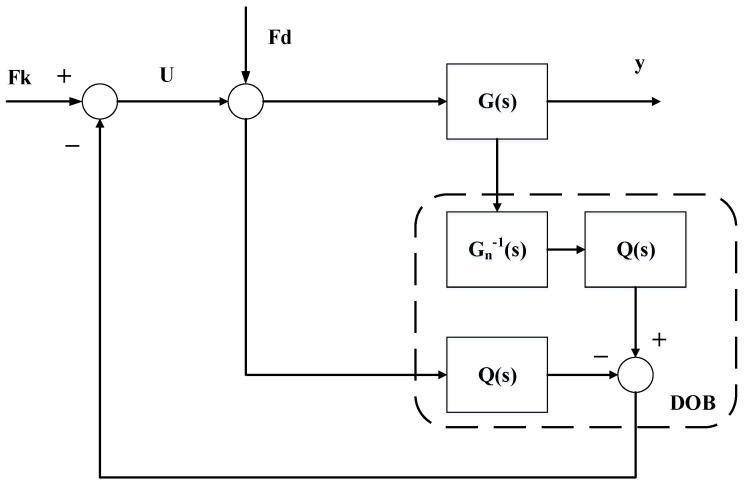
Block diagram of disturbance observer (DOB).

To improve the suppression of the disturbances transmitted from the base platform, a DOB was added to this study; its block diagram is shown below in Figure 4.

In Figure 4, $F_{k}$ is the force calculated by the LQR controller K, which is obtained from the previous section; $F_{d}$ is the disturbance force transmitted from the base platform; G(s) represents the real uncertainties of the plant of the system; $G_{n}(s)$ is the nominal model; Q(s) is the selected low-pass filter; and U is the final output control voltage. The measured signal y of the system contains the joint effects of the control and disturbance forces for the plant G(s), which can both be considered control inputs in the disturbance observer. The equivalent disturbance is estimated by designing the nominal model $G_{n}(s)$ and the filter Q(s), and that is imported into the controller as compensation to achieve the suppression of the disturbance. Using Equation (3), the transfer function of the acceleration of the payload platform and the applied combined force can be written as:

(15) $$G(s)=\frac{s^{2}x(s)}{F_{c}(s)+F_{d}(s)}=\frac{s^{2}x(s)}{F(s)}=\frac{s^{2}}{ms^{2}+cs+k},$$

In principle, a suitable low-pass filter Q(s) is required to ensure that the overall transfer function is positive and thereby enable the DOB to estimate the direct disturbance by inverse nominal model calculation. The order of the filter should be suitably designed because it affects the stability of the DOB and is not conducive to real-time control. In this study, the force balance equation is satisfied as a positive-definite inverse model function; so, the low-pass filter can be chosen more conservatively as only a first-order filter:

(16) $$Q(s)=\frac{1}{\tau s+1},$$

where τ is the time constant of the filter. The DOB can have a suppression effect in a wide frequency range; it was designed with τ=0.001.

Generally, it is difficult for the parameters of the designed $G_{n}(s)$ to be the same as those in the real plant G(s). However, according to the conclusions mentioned by Hyungbo, robust stability can be achieved for bounded uncertainty models if the uncertainty system is a minimum phase system, provided that the outer-loop controller is stable [34]. The real uncertainty model G(s) used in this study had open-loop zeros in the left half-plane of s, i.e., which are minimum-phase systems. Additionally, the outer-loop LQR controller designed could remain stable; the effects caused by parameter uncertainty could be neglected.

### 3.3. Simulation

**Figure 5:**
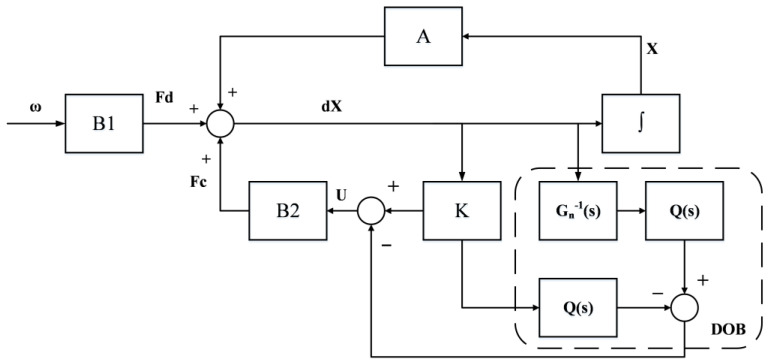
Control system with linear quadratic regulator (LQR) and DOB. The system state differential dX is used as the control input. With the DOB, the disturbance force is estimated. The result of the LQR controller K is combined with the compensation of the DOB to become the output control voltage U. By the voice coil motor, the active control force $F_{c}$ is generated to suppress the vibration.

**Figure 6:**
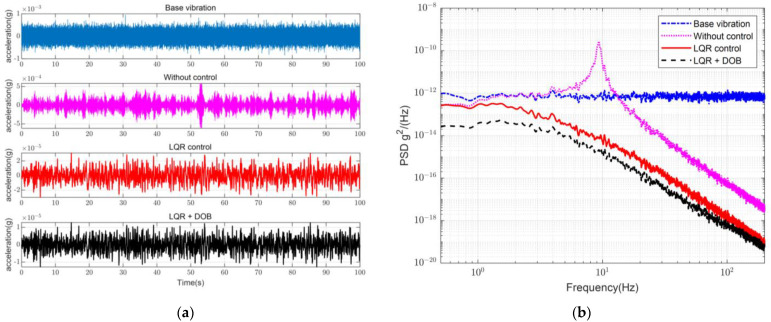
Simulation curves of active vibration isolation control using different control approaches. (**a**) Vibration signal in time domain; (**b**) Power spectral density (PSD).

The control loop is shown in Figure 5. The system was subjected to disturbance forces transmitted from the base platform and control forces driven by the VCM, where the control forces were obtained through a combination of disturbances calculated by the LQR controller and DOB. Simulation experiments were conducted on a single vibration isolation module to compare the dynamic characteristics of the passive isolator and active control. The simulated physical parameters were similar to those of the real system. The vibration signal in the time domain and PSD obtained from the experimental results are shown in Figure 6.

Regarding the white noise disturbances, the vibration was amplified at the resonance peak that existed owing to the system structure and was then suppressed in the frequency range. Regarding the state-differential feedback control with the LQR, the effect of the resonance peak was significantly eliminated, and the start frequency of the suppression was advanced to 1 Hz, providing the system with a certain degree of vibration isolation over the entire frequency range. With the addition of the DOB section, the vibration isolation capability of the low-frequency region was further improved.

## 4. Experimental Validation

### 4.1. Experimental System

**Figure 7:**
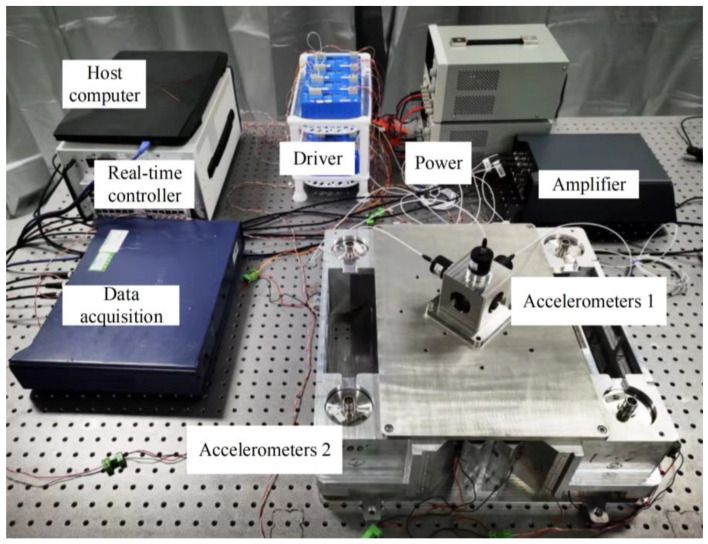
Schematic diagram of experimental system.

The active vibration isolation system used in this study is shown in Figure 7. The accelerometer measurements in the eight vibration isolation modules were collected by the real-time simulator analog to digital (AD) and outputted by the digital to analog (DA) to drive the VCMs. The vibration out-loop data were obtained by monitoring the sensor measurements and data acquisition and used to observe any variations. Moreover, the host computer could obtain in-loop data and adjust the controller parameters in real time. The controller program was written in LabVIEW 2020. The physical parameters of each part of the system are listed in the Table 1.

### 4.2. Analysis of Experimental Results

**Figure 8:**
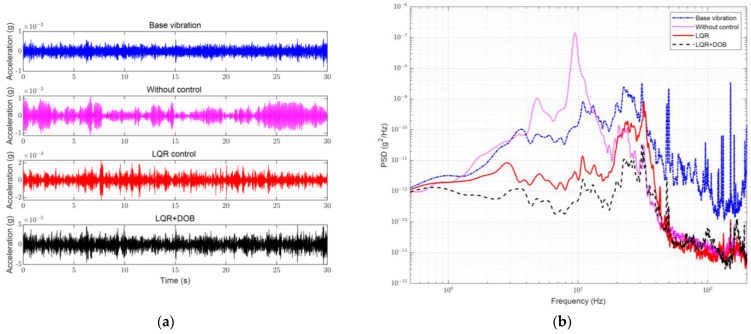
Performance of active vibration isolation control with different controls in Z-direction. (**a**) Acceleration in time domain; (**b**) Measured PSD.

In this study, the experiments were carried out on an optical platform in the laboratory; the environmental noise was regarded as the source of the disturbance. An active vibration isolation system was tested for vibration acceleration along the Z-, X-, and Y- directions. A comparison of the experimental results for the base vibration without control, with the LQR control, and with the LQR + DOB is shown in Figure 8.

Figure 8 shows the comparison of the vibration accelerations in the Z-direction (direction of gravity). The time-domain amplitude of the disturbance source in this direction was approximately 1 mg, and the vibration was most pronounced in the frequency domain at 25 Hz, owing to the characteristics of the optical platform. The time-domain amplitude of the vibration was not significantly decayed without control because of the system’s low damping and high resonance peak. However, starting at 20 Hz, the vibration was significantly attenuated, and the maximum effect of attenuation was reached at 70 Hz, which proved that passive vibration isolation was significant in the high-frequency range. With the active control under the LQR, the amplitude of the time-domain signal was suppressed to be within 0.2 mg; in the frequency domain, the low-frequency band signal was actively isolated at 1 Hz. The passive resonance peak was suppressed, while the high-frequency band also retained its performance. After adding the DOB, the amplitude of the time-domain signal decayed to 50 µg; and the low-frequency vibration, including the resonance peak, was further suppressed. The maximum rejection ratio of the PSDs within 10 Hz was increased by 9.6 dB, and the starting vibration isolation frequency also expanded downward.

**Figure 9:**
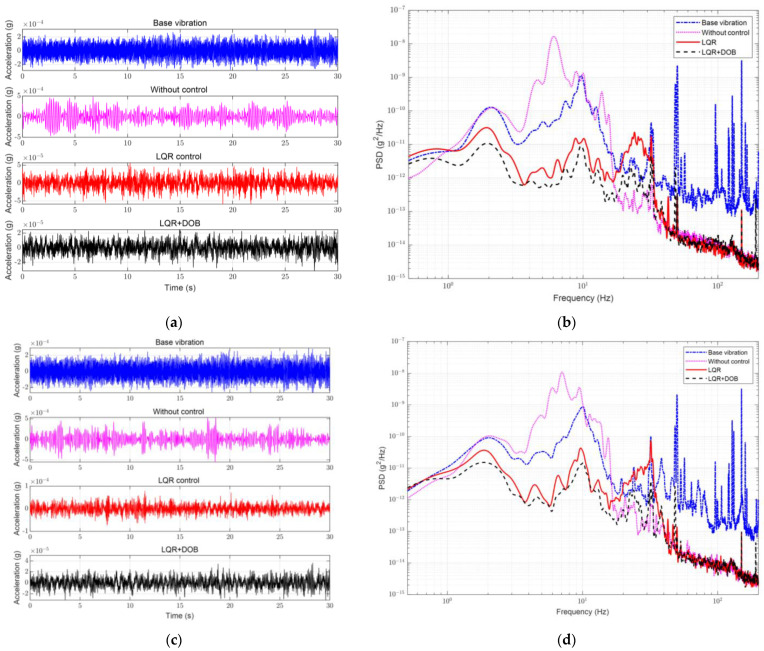
Performance of active vibration isolation control with different controls in X- and Y-directions. (**a**) Acceleration in time domain in X-direction; (**b**) Measured PSD in X-direction; (**c**) Acceleration in time domain in Y-direction; (**d**) Measured PSD in Y-direction.

Compared with that under the Z-direction, the disturbance sources under the X- and Y- directions were weaker as shown in Figure 9. The amplitudes of the time-domain signals in the horizontal directions were no more than 0.2 mg, and the frequency domain was mainly concentrated at 10 Hz. Additionally, the resonant frequencies in the horizontal direction were noticeably lower at 6.4 and 6.7 Hz. Regarding the active vibration isolation, the LQR control still performed satisfactorily for low-frequency band vibrations, including the resonance peak. Adding the DOB also further optimized the overall low-frequency-band attenuation rate, and the maximum rejection ratio of the PSDs within 10 Hz in the X- and Y-directions was improved by 8.2 and 6.1 dB, respectively. The root-cumulative PSD within 200 Hz under different control conditions in the three directions is presented in Table 2.

The controller of the LQR + DOB combination had an obvious improvement in the vibration isolation index. Compared with the base disturbance source, the controller designed in this study could be reduced by 1.34 × 10^−4^ g, 7.16 × 10^−5^ g, and 6.87 × 10^−5^ g in the Z-, X-, and Y- directions, respectively, in the root-cumulative PSD. The corresponding vibration isolation suppression rates were achieved at 91.9, 90.9, and 88.7%. Considering the optical reference cavity designed by the NPL, the vibration sensitivity in all three directions was considered to have a maximum value of 2.5 × 10^−11^ g. According to Equation (1), the frequency stability created by the vibration could be constrained to 3.22 × 10^−17^ g using active vibration isolation control.

## 5. Conclusions

In this study, a state-differential feedback controller with a DOB-based LQR is proposed to meet the vibration isolation requirements of an optical reference cavity. The LQR controller was designed using the LMI method, and a DOB was developed based on the inverse function of the nominal model to estimate the disturbance force from the base platform. The simulation and experimental results showed that the controller had a significant suppression effect on the vibration and that adding the DOB could further improve the suppression effect. The experimental results also demonstrated that the maximum suppression ratios of the PSD in the Z-, X-, and Y- directions within 10 Hz were significantly improved. Compared to the disturbance source noise, the suppression rates of the root-cumulative PSD in the three directions were significant. The comprehensive vibration isolation performance met the frequency-stability standard of the laser. The effectiveness and feasibility of the controller for the vibration isolation of the optical reference cavity were verified.

Active control of the disturbance source allows the system to achieve advantageous vibration isolation performance. For microvibration, the measurement noise from the sensor is also a significant factor in the performance of the system. How to further reduce the noise in the low-frequency band deserves more attention for future research.

## Figures and Tables

**Table 1:** Physical parameters of experimental system.

Parameter	Value	Unit
Mass m	25	Kg
Stiffness k	88,000	N/m
Damping c	17.6	Ns/m
Resonant frequency $\omega_{z}$	10.12	Hz
Resonant frequency $\omega_{x}$	6.38	Hz
Resonant frequency $\omega_{y}$	6.71	Hz
Sensor sensitivity $K_{S}$	1000	V/g
Gain of VCM $K_{V}$	1.2	N/A
Gain of Driver $K_{A}$	0.2	A/V

**Table 2:** Root-cumulative PSD in the three directions.

Direction	Base Vibration (g)	LQR Control (g)	LQR + DOB (g)
Z	1.46 × 10^−4^	4.61 × 10^−5^	1.17 × 10^−5^
X	7.88 × 10^−5^	1.48 × 10^−5^	7.16 × 10^−6^
Y	7.75 × 10^−5^	1.70 × 10^−5^	8.76 × 10^−6^

## Data Availability

Not applicable.
